# Monocytes as Regulators of Inflammation and HIV-Related Comorbidities during cART

**DOI:** 10.1155/2014/569819

**Published:** 2014-06-12

**Authors:** Joshua J. Anzinger, Tiffany R. Butterfield, Thomas A. Angelovich, Suzanne M. Crowe, Clovis S. Palmer

**Affiliations:** ^1^Department of Microbiology, The University of the West Indies, Kingston, Jamaica; ^2^Centre for Biomedical Research, Macfarlane Burnet Institute for Medical Research and Public Health, GPO Box 2284, Melbourne, VIC 3001, Australia; ^3^School of Applied Sciences, RMIT University, Melbourne, VIC 3000, Australia; ^4^Department of Medicine, Monash University, Melbourne, VIC 3800, Australia; ^5^Department of Infectious Diseases, Monash University, Melbourne, VIC 3800, Australia

## Abstract

Combined antiretroviral therapy (cART) extends the lifespan and the quality of life for HIV-infected persons but does not completely eliminate chronic immune activation and inflammation. The low level of chronic immune activation persisting during cART-treated HIV infection is associated with the development of diseases which usually occur in the elderly. Although T-cell activation has been extensively examined in the context of cART-treated HIV infection, monocyte activation is only beginning to be recognized as an important source of inflammation in this context. Here we examine markers and sources of monocyte activation during cART-treated HIV infection and discuss the role of monocytes during cardiovascular disease, HIV-associated neurocognitive disorder, and innate immune aging.

## 1. Introduction 

The introduction of combination antiretroviral therapy (cART) has dramatically increased survival of HIV-infected persons [1, 2]. Once only widely available in high-income countries, access to cART has steadily increased over the last decade in low- and middle-income countries where the majority of HIV-infected persons live. In 2011, for the first time cART became available to the majority (54%) of HIV-infected persons eligible for treatment in low- and middle-income countries, with the percentage of cover expected to continue to increase in the coming years [3].

Due to improved access and adherence to cART, it is predicted that most HIV-infected persons worldwide will live longer, healthier lives. However, recent observations have identified that effectively treated HIV-infected persons do not live as long as age-matched HIV-uninfected persons [4]. The cause of death for most HIV-infected persons has changed from AIDS-related opportunistic infections to chronic diseases with an inflammatory pathogenesis usually associated with the elderly [5]. The premature onset and increased risk of these inflammatory age-related diseases are associated with low levels of chronic immune activation that persist during cART treatment, a process that is believed to contribute to serious non-AIDS events (SNAEs). While most research examining chronic immune activation has focused on activation of T cells, the role of activated monocytes in promoting chronic inflammation during cART-treated HIV infection has been less thoroughly investigated.

Recent studies indicate that inflammatory mediators produced by monocytes, but not T-cell activation, predict SNAEs in virologically suppressed HIV-infected persons treated with cART [6, 7], demonstrating the important role of monocyte activation during cART-treated HIV infection. In these studies, the level of IL-6, a cytokine produced at high levels by monocytes that can also be produced at lower levels by other cell types in certain circumstances [8], was associated with increased odds of SNAE and death but not the percentage of activated CD4 and CD8 T cells (those expressing CD38 or CD38 and HLA-DR). These recent studies suggest that monocytes are a major source of inflammation in virologically suppressed persons treated with cART.

## 2. HIV Associated Comorbidities in the cART Era

Many of the diseases observed in cART-treated HIV-infected persons show similarities with chronic inflammatory disorders and diseases that predominantly occur in the elderly, such as cardiovascular disease (CVD), neurocognitive disorders, non-AIDS cancers, osteoporosis, and frailty. While the mechanisms defining these similarities have not been elucidated, it is believed that chronic inflammation, which remains a constant between these diseases, contributes to SNAEs caused by these diseases. Monocytes are chronically activated during HIV infection, and a large body of evidence now suggests that activated monocytes in the context of HIV infection are major mediators for the development of CVD, neurocognitive disorder, and aging of the innate immune system.

### 2.1. Cardiovascular Disease

CVD has emerged as one of the leading causes of death among HIV-infected persons in the cART era [5, 9]. HIV-infected persons are at an increased risk for developing CVD compared to HIV-uninfected controls, with HIV-infected cART-treated persons having a greater risk of developing CVD than treatment-naïve HIV-infected persons [10, 11]. The monocyte markers CD11b and CX3CR1 are associated with subclinical atherosclerosis in HIV-infected persons treated with cART [12], indicating an important role for monocytes in promoting CVD for these persons.

CVD-related deaths in the general population as well as in HIV-infected persons are attributed to underlying atherosclerosis, a disease in which monocytes play a central role. One of the earliest events in the development of atherosclerosis is mediated by activation of endothelial cells lining the wall of blood vessels [13], a process that is promoted by HIV infection [14]. Endothelial cell dysfunction leads to the recruitment and accumulation of circulating monocytes in the subendothelial lining. Once in the vessel wall, monocytes differentiate into macrophages that produce a number of proinflammatory mediators, some of which recruit additional monocytes to the lesion [15–19]. Macrophages in the vessel wall take up high levels of the cholesterol-rich particle low-density lipoprotein (LDL), causing cholesterol to accumulate under conditions of elevated LDL [20]. The accumulation of cholesterol in macrophages reduces their ability to emigrate out of atherosclerotic plaques [21] and can induce macrophage necrosis, resulting in a cholesterol-rich necrotic core that is prone to rupture and causes a deleterious clinical event [22, 23]. Elevated plasma LDL strongly predicts coronary artery disease and is the primary target for cholesterol-lowering therapies [24]. Treatment of HIV infection with cART may cause elevations in LDL-cholesterol [25–27] and can be associated with increased risk of cardiovascular disease compared to treatment-naïve and HIV-uninfected persons [10]. Certain protease inhibitors and nonnucleoside reverse transcription inhibitors are associated with elevated LDL-cholesterol, with some protease inhibitors associated with greater LDL-cholesterol elevations and risk of cardiovascular disease [10, 28, 29].

High-density lipoprotein (HDL) can remove cholesterol from macrophages using cell surface cholesterol transporters. However, during conditions of elevated LDL, as observed during cART-treated HIV infection [25–27], the rate of LDL-derived cholesterol accumulation in macrophages can be greater than cholesterol removal by HDL, resulting in net cholesterol accumulation in the vessel wall. Further favoring macrophage cholesterol accumulation, removal of macrophage cholesterol by HDL may be hampered during HIV infection, as cholesterol removal from HIV-infected human macrophages by HDL is impaired due to decreased levels and functionality of the cholesterol transporter ABCA1 [30]. Although HIV-infected macrophages within atherosclerotic plaque have been identified in HIV-infected individuals treated with cART [30], it is unclear if ABCA1-mediated cholesterol efflux from macrophages is also impaired in vivo. SIV-infected macaques fed an atherogenic diet have dysfunctional HDL that is likely mediated by nef downregulation of macrophage and liver ABCA1 [31], suggesting that inhibition of ABCA1-mediated cholesterol efflux from macrophages also occurs in vivo.

Most deaths attributable to atherosclerosis are due to thrombus formation. During cART-treated HIV infection, monocytes are chronically activated and can produce factors that stimulate thrombosis. When monocytes are activated in vitro with lipopolysaccharide (LPS), they produce microparticles that stimulate formation of the clotting factor fibrin [32]. Tissue factor is a clotting factor expressed on leukocytes that can also initiate thrombus formation. HIV-infected persons show an association between monocyte expression of tissue factor and the coagulopathy marker D-dimer [33, 34], providing evidence that monocytes may facilitate a prothrombotic environment. When peripheral blood monocyte subsets of HIV-uninfected and cART-treated HIV-infected patients with viral loads <400 copies/mL are compared, an increased percentage of nonclassical and intermediate monocytes expressing tissue factor are observed in HIV-infected patients [35]. In HIV-uninfected individuals with coronary heart disease, both nonclassical and intermediate monocyte subsets show increased platelet aggregation compared with healthy controls [36], demonstrating the prothrombotic role of these monocyte subsets.

Macrophages take up high levels of glucose in atherosclerotic plaques to facilitate the production of proinflammatory mediators [37]. In vivo plaque-resident macrophages take up the glucose imaging agent ^18^fluorodeoxyglucose, a process used to identify atherosclerotic plaques with an inflammatory phenotype [37]. In HIV-infected patients treated with cART, ^18^fluorodeoxyglucose accumulates at higher levels in the ascending aorta and carotid arteries compared to HIV-uninfected controls [38, 39], with aortic uptake of ^18^fluorodeoxyglucose associated with the macrophage specific marker soluble CD163 [39]. These studies suggest that glucose uptake by macrophages may contribute to the increased cardiovascular disease risk associated with HIV-infected patients treated with cART. We recently identified intermediate (CD14^++^CD16^+^) monocytes expressing glucose transporter 1 (Glut1) as being significantly elevated in blood from HIV-infected individuals compared with HIV-uninfected individuals, regardless of cART treatment status [40]. These Glut1^+^ intermediate monocytes are activated [40], take up high levels of glucose [40], and retain Glut1 expression when differentiated into macrophages (Palmer CS and Crowe SM, unpublished observation). As Glut1 mRNA levels in atherosclerotic plaques predict accumulation of ^18^fluorodeoxyglucose [41], our results suggest that Glut1^+^ intermediate monocytes may be important mediators of cardiovascular disease.

### 2.2. HIV-Associated Neurocognitive Disorder

HIV-associated neurocognitive disorder (HAND) is a term that encompasses varying degrees of neurological impairment, from asymptomatic neurocognitive impairment to mild neurocognitive impairment and the most severe, HIV-associated dementia (HAD). Although cART has substantially reduced severe neurological impairment [42], milder forms of HAND continue to occur in up to fifty percent of HIV-infected persons in the cART era [42–46]. This neurological impairment persists despite virologically suppressive cART treatment and can lead to non-AIDS neuropsychiatric events even with CD4 counts >500 cells/mm^3^ [47].

The main HIV-infected cell type in the brain is macrophages [48, 49]. As the blood brain barrier is a highly selective barrier for solutes to traverse [50], it was initially proposed [51] and is now widely believed that HIV-infected monocytes traversing the blood brain barrier are a major source of HIV found in the brain [52]. Macrophages are in close proximity to the vasculature, and fluorescently labeled monocytes injected into acutely SIV-infected rhesus macaques accumulate in the brain and coincide with SIV detection in the brain [53].

Monocytes can remain productively infected with HIV during cART treatment [54–56] and are likely to represent a source of HIV found within the brain of HIV-infected persons treated with cART. HIV-infected monocytes that enter the brain are thought to give rise to perivascular macrophages which are commonly infected with HIV. Microglia are less commonly infected than perivascular macrophages and arise from different cell precursors [49, 57–59]. The level of HIV DNA in monocytes, but not plasma viral load or CD4 count, is associated with HAND for HIV-infected persons before and after cART treatment [60], with the association persisting at 3.5 years after cART initiation [61]. It is likely that some of these HIV-infected monocytes harboring HIV DNA could cross the blood brain barrier, contributing to the persistent presence of HIV-infected cells in the brain. As HIV-infected persons treated with cART show preferential HIV infection in CD16^+^ proinflammatory monocytes compared to CD16^−^ monocytes [62], CD16^+^ monocytes could be a source of HIV-infected monocytes in the brain. In these experiments, T cells from PBMC were removed by magnetic beads prior to monocyte isolation, making it unlikely that T-cell contamination could explain the presence of HIV in monocytes. Although several studies suggest that HIV-infected monocytes can introduce HIV into the brain, this has not been conclusively demonstrated.

After HIV-infected persons are treated with cART, immune activation is decreased considerably but remains elevated compared with HIV-uninfected persons [63]. Activated macrophages produce the monocyte-derived immune activation marker neopterin, a molecule thought to participate in maintaining reactive oxygen and nitrogen products produced by macrophages [64, 65]. cART-treated HIV-infected persons with complete viral suppression for ≥3.5 years have elevated levels of cerebral spinal fluid neopterin compared with HIV-uninfected controls [66], indicating persistent low levels of macrophage activation in the central nervous system. Infection of monocytes with HIV or stimulation by gp120 causes monocytes to produce neurotoxic factors that interact with neuronal N-methyl-D-aspartate receptors [67, 68]. Neuronal stimulation of N-methyl-D-aspartate receptors can result in neuron death by apoptosis or necrosis [69]. In addition, HIV production by macrophages in the brain also results in neuronal toxicity, as several HIV components can interact with neurons and cause toxicity [70–72].

### 2.3. Innate Immune Aging

Chronic immune activation causes monocytes to become dysfunctional and share characteristics of monocytes from the elderly. In a recent study by Martin and colleagues, proinflammatory cytokines produced by monocytes and markers of immune aging were shown to be elevated in age-matched HIV-infected women (87% of whom were receiving cART treatment) compared to HIV-uninfected women [73]. The levels of these cytokines and markers of immune aging were comparable to the levels seen in HIV-uninfected women 10.6–14.5 years older, demonstrating that HIV-infected persons display an aged phenotype [73].

In young HIV-infected women there are an increased proportion of CD16^+^ proinflammatory monocytes, similar to that observed in HIV-uninfected women 10.6 years older [73]. Young HIV-infected males, both treated and untreated, have increased plasma levels of the inflammatory biomarkers neopterin, sCD163, and CXCL10 when compared to age-matched HIV-uninfected males [74]. The levels of these inflammatory molecules in young treated and untreated HIV-infected males are comparable to the levels seen in older HIV-uninfected men, indicating that accelerated innate immune aging induced by HIV infection is not restored by cART [74]. Similar to what is seen in the elderly, monocytes in blood from young HIV-infected men have impaired phagocytosis and shortened telomeres implicating accelerated innate immune aging that might underlie the dysfunction of monocytes in the setting of HIV infection [74]. The “aging” of monocytes during HIV infection, even during virologically suppressive cART treatment, is likely to contribute to the development of premature age-related diseases.

## 3. Monocyte Parameters of Systemic Inflammation

The detection of biological markers that identify cART-treated HIV-infected individuals with increased risk of comorbid disorders is useful for the management of these disorders. Recent work has identified biological markers associated with monocytes and/or macrophages that predict non-AIDS mortality.

### 3.1. Interleukin-6

Interleukin-6 (IL-6) is a proinflammatory cytokine produced by monocytes and macrophages during trauma, infection, and stress that instigate acute-phase protein production and inflammation [8]. Both untreated and cART-treated HIV-infected persons have elevated levels of IL-6 [63, 75], with elevated IL-6 levels associated with increased risk of all-cause mortality and death due to CVD in HIV-infected persons [34]. Monocytes from HIV-infected persons at risk for CVD produce higher levels of IL-6 compared with HIV-uninfected persons at risk for CVD [76], providing a potential explanation for the inflammatory pathogenesis and related increased CVD risk associated with HIV infection. The association of IL-6 with increased risk of CVD for HIV-infected persons is independent of other risk factors and higher levels of IL-6 are associated with a hazard ratio higher than for levels of other inflammatory markers such as hsCRP and D-dimer [77]. In addition to CVD, persons with HAND have elevated IL-6 levels in cerebral spinal fluid that remains elevated 12 weeks after initiation of cART [78]. The elevated levels of IL-6 observed during HIV infection are also observed in the elderly [79], suggesting that low levels of chronic inflammation and chronic production of IL-6 could lead to immunosenescence observed during normal aging as well as in chronic HIV infection. As lymphocytes are activated by IL-6 [80], it is possible that chronic stimulation with IL-6 could lead to immunosenescence. Consistent with this idea, elderly persons with elevated IL-6 levels have decreased responsiveness to vaccination compared to elderly persons with lower levels of IL-6 [81]. However, it is currently unresolved whether IL-6 is a cause or consequence of immunosenescence.

### 3.2. Soluble CD14

CD14 is a coreceptor expressed predominantly on monocytes and macrophages that together with TLR4 recognize LPS and other pathogen-associated molecular pattern molecules. After activation, monocytes produce soluble CD14 (sCD14) by enzymatic shedding of CD14 from the plasma membrane [82]. Plasma levels of sCD14 are significantly elevated in HIV-infected persons, regardless of cART treatment status, compared with healthy controls [83, 84]. The plasma level of sCD14 in HIV-infected persons is an independent predictor of mortality and correlates with levels of the inflammatory molecules IL-6, CRP, serum amyloid A, and D-dimer [85]. Plasma sCD14 levels in HIV-infected persons also correlate with carotid artery intima-media thickness (a measurement of atherosclerosis) independent of HIV infection and type of antiretroviral therapy [86]. In addition to cardiovascular disease, increased plasma levels of sCD14 have been shown to be associated with neurological impairment in HIV-infected individuals as assessed by formal neurological testing and evaluations [75]. Although sCD14 is produced by activated monocytes, hepatocytes also secrete sCD14 as an acute-phase protein [87]. Therefore, measurement of plasma sCD14 may not be an exclusive representation of the levels of monocyte activation, a factor that should be considered when utilizing this plasma marker.

### 3.3. Soluble CD163

CD163 is a hemoglobin scavenger receptor expressed exclusively on monocytes and macrophages. Activation of monocytes and macrophages with LPS and other stimuli causes CD163 to be shed from the cell surface in a soluble form, referred to as soluble CD163 (sCD163) [88]. As sCD163 is shed only from mononuclear phagocytes, it is a specific activation marker for these cells. Although sCD163 is associated with monocyte activation and inflammatory diseases, it has anti-inflammatory effects and is believed to be involved in resolving inflammation [88]. Compared with HIV-seronegative controls, plasma sCD163 is elevated in chronically HIV-infected persons before ART and is reduced 3 months after ART, but at levels that are elevated compared to controls [89]. Plasma sCD163 is also increased during acute HIV infection compared to HIV-seronegative controls though at lower levels than chronic infection [89]. In acutely infected patients treated with cART for three months, sCD163 levels are similar to those in HIV-seronegative controls [89], suggesting that early cART initiation can limit mononuclear phagocyte activation.

Elevated levels of sCD163 are observed in several comorbidities associated with cART-treated HIV infection. Plasma sCD163 is associated with an increased prevalence of atherosclerotic plaques in cART-treated HIV-infected persons with undetectable HIV RNA, a relationship that is not observed in HIV-negative controls matched for cardiovascular risk factors [90]. The authors suggest that activation of mononuclear phagocytes during HIV infection could cause a unique atherosclerotic plaque phenotype not observed in HIV-uninfected persons. In support of this, young (23–32 years old) HIV-infected persons show a unique atherosclerotic plaque phenotype that resembles a phenotype observed in patients that rejected cardiac transplant [91]. cART-treated HIV-infected patients with HAND have elevated levels of plasma sCD163 compared with HIV-infected controls without HAND [92], indicating an important role of activated mononuclear phagocytes during HAND. Finally, elevated levels of sCD163 occur at an earlier age in HIV-infected women than in uninfected women [73], suggesting that chronic mononuclear phagocyte activation is a mediator of immunosenescence.

## 4. Sources of Monocyte Activation

**Figure 1:**
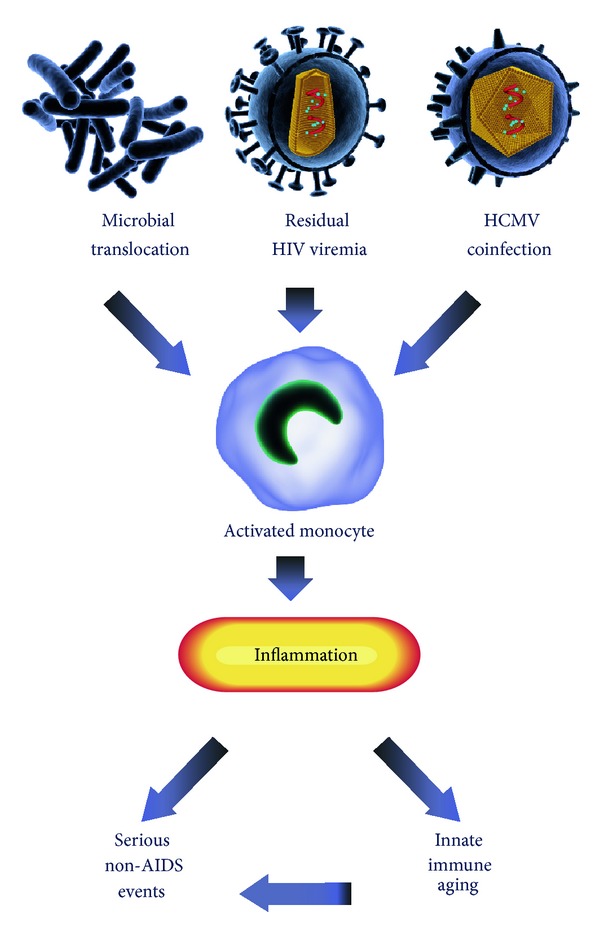
Microbial translocation, residual HIV replication, and coinfections such as HCMV cause persistent monocyte activation and contribute to chronic inflammation in HIV+ individuals receiving antiretroviral therapy. This results in innate immune aging and may influence the development of age-related diseases. Image created by http://nice-consultants.com/.

The source of chronic inflammation observed during cART-treated HIV infection has been an area of intense research in recent years, as it is believed to be the underlying cause for the increased risk of SNAEs that are progressively seen in clinics caring for HIV-infected persons treated with cART. Three major mechanisms have been proposed to explain the persistently high levels of inflammation in HIV-infected individual on antiretroviral treatment: (1) increased microbial translocation through the compromised intestinal mucosa, (2) residual HIV viremia, and (3) coinfection with human cytomegalovirus (HCMV) and other pathogens. Each of these mechanisms is associated with monocyte activation that is likely to contribute directly to SNAEs or indirectly by induction of innate immune aging (Figure 1). Microbial translocation, residual HIV viremia, and coinfection with pathogens may be codependent processes. For example, coinfecting pathogens and products from microbial translocation could activate HIV-infected cells to produce low levels of HIV that contribute to residual viremia present in HIV-infected persons treated with cART, and residual viremia and coinfecting pathogens could contribute to damage of the intestinal mucosa, enhancing microbial translocation.

### 4.1. Microbial Translocation

During acute HIV infection there is a dramatic depletion of gut CD4 T cells, resulting in increased permeability of the gut mucosal barrier that persists during chronic untreated HIV infection and also during cART treatment [93, 94]. This increased gut permeability allows bacterial components such as the Gram-negative bacterial cell wall component LPS to become elevated in the plasma of both untreated and cART-treated HIV-infected persons compared to HIV-uninfected controls [93]. Elevated plasma levels of LPS during HIV infection results in increased plasma sCD14, signifying that circulating monocytes are chronically activated by LPS [93]. This increase in sCD14 is positively correlated with LPS levels, suggesting that monocyte activation by LPS is likely responsible for sCD14 production [93]. Activation of monocytes by LPS also causes increased levels of sCD163 [88], a mononuclear phagocyte activation marker that is elevated in untreated and cART-treated HIV-infected persons compared to HIV-uninfected controls [89]. These observations indicate that microbial translocation is likely to be a key inducer of monocyte activation and chronic low level systemic inflammation observed in individuals infected with HIV.

Activation of monocytes by LPS may be exacerbated due to alterations in HDL levels that are associated with HIV infection. Plasma lipoproteins bind LPS, the majority of which is bound to HDL [95]. HDL binding of LPS neutralizes the stimulatory activity of LPS towards monocytes in vitro [96], and LPS treatment of persons with low HDL levels is associated with higher levels of inflammatory mediators compared to persons with higher HDL levels treated with LPS [97]. These data indicate that HDL can limit inflammation induced by LPS. As cART-treated HIV-infected men and certain cART-treated HIV-infected women have decreased HDL-cholesterol levels [26, 98], the level of neutralized plasma LPS may be limited in these persons.

In addition to the activation of circulating monocytes, microbial translocation induces the accumulation of proinflammatory, functionally impaired macrophages within the subepithelium of the gut in untreated HIV-infected individuals [99]. These macrophages show increased expression of proinflammatory cytokines and chemokines and are unable to phagocytose microbes or microbial products [99]. The inflammatory characteristics of these macrophages may exacerbate microbial translocation since the proinflammatory cytokines they produce can increase gut epithelial permeability and thereby allow microbes and microbial products to cross the mucosal barrier [99].

### 4.2. Residual HIV Viremia

The introduction of cART has resulted in frequent reduction of HIV viremia to undetectable levels as assessed by conventional techniques. The SMART study highlighted that intermittent cART resulted in elevated inflammation and higher mortality and morbidity among HIV-infected persons who ceased therapy when compared to those receiving continuous cART [34, 100]. This underscores the significance of suppressed viral replication and repression of inflammation in the management of persons infected with HIV [100].

Ultrasensitive assays capable of detecting HIV in plasma at 1 copy/mL have demonstrated that low levels of HIV viremia continue to occur in individuals with virologic suppression (i.e., <50 copies/mL) during cART [101–103]. Raltegravir intensification, introduced in patients to suppress residual viremia, resulted in lower plasma levels of the inflammatory procoagulant marker D-dimer in some treated HIV-infected individuals compared to patients receiving placebo, illustrating a potential link between low level viral replication and inflammation [104]. However, residual viremia is unaffected by raltegravir intensification [105]. It therefore remains unclear if the residual viremia that occurs in virologically suppressed HIV-infected persons treated with cART is due to HIV replication or production (i.e., generation of new viruses without completion of the replication cycle) [106]. Regardless of the mechanism of residual HIV viremia, it is likely to be a chronic source of monocyte activation because many components of HIV induce monocytes to produce proinflammatory molecules [107–109].

### 4.3. Coinfections

Most HIV-infected persons are latently infected with HCMV and are able to effectively control this virus [2, 110, 111]. However, it is now clear that HIV-infected persons invest a considerable immune response to limit pathogenesis of HCMV even when HIV replication is controlled by cART. For example, the percentage of HCMV-specific CD8 T-cell clones in HIV-infected persons treated with cART is twice that of HIV-uninfected persons [112], indicating an important role of the cellular immune response in controlling HCMV replication. Although HCMV has a broad cell tropism, monocytes are believed to be important at disseminating HCMV to tissue as they migrate with latent virus and produce virus during differentiation [113, 114]. When infected with HCMV, monocytes become activated and proinflammatory genes are upregulated [115, 116].

HIV-infected persons treated with cART are also commonly infected with herpesviruses other than HCMV that can also establish latency. Epstein-Barr virus (EBV), human herpesvirus 8 (HHV-8), and herpes simplex virus type 1 are more commonly detected in the saliva of cART-treated HIV-infected persons compared to HIV-uninfected controls [117]. Monocytes can be infected with EBV which causes reduced phagocytic functionality [118, 119], and stimulation of monocyte TLR2 by EBV promotes induction of cytokine secretion [119]. HHV-8 can infect monocytes and macrophages and establish productive infection when stimulated with inflammatory cytokines [120–122], and HHV-8 infection induces upregulation of monocyte TLR3 and production of inflammatory cytokines [123]. HSV-1 can also infect monocytes and macrophages, which produce inflammatory cytokines when exposed to HSV-1 [124, 125]. Though not directly examined, herpesvirus coinfection is therefore likely to be a source of chronic monocyte activation in the context of cART-treated HIV infection.

## 5. Conclusion

With the majority of cART-eligible HIV-infected persons now receiving treatment, SNAEs have increased and are one of the greatest health concerns for HIV-infected persons. In HIV-infected persons treated with cART, monocytes are an important source of proinflammatory mediators associated with cardiovascular disease, HIV-associated neurocognitive development, and innate immune aging. It remains to be determined if monocytes are also mediators of other premature age-related diseases such as non-AIDS cancer and liver diseases that cART-treated HIV-infected persons are at an increased risk for developing. With sources of monocyte activation and identification of monocyte activation pathways emerging in recent years, therapeutically targeting sources and pathways of monocyte activation could be a useful strategy to limit immune activation associated with the premature development of age-related diseases for HIV-infected persons treated with cART.

## References

[1] Crum NF, Riffenburgh RH, Wegner S (2006). Comparisons of causes of death and mortality rates among HIV-infected persons: analysis of the pre-, early, and late HAART (highly active antiretroviral therapy) eras. *Journal of Acquired Immune Deficiency Syndromes*.

[2] Palella FJ, Delaney KM, Moorman AC (1998). Declining morbidity and mortality among patients with advanced human immunodeficiency virus infection. HIV Outpatient Study Investigators. *The New England Journal of Medicine*.

[3] UNAIDS World AIDS Day Report.

[4] Wada N, Jacobson LP, Cohen M, French A, Phair J, Muñoz A (2013). Cause-specific life expectancies after 35 years of age for human immunodeficiency syndrome-infected and human immunodeficiency syndrome-negative individuals followed simultaneously in long-term cohort studies, 1984–2008. *American Journal of Epidemiology*.

[5] Rodger AJ, Lodwick R, Schechter M (2013). Mortality in well controlled HIV in the continuous antiretroviral therapy arms of the SMART and ESPRIT trials compared with the general population. *AIDS*.

[6] Tenorio AR, Zheng Y, Bosch RJ Soluble markers of inflammation & coagulation, but not T-cell acivation, predict non-AIDS defining events during suppressive antiretroviral therapy (ART).

[7] Hunt PW, Sinclair E, Rodriguez B Gut epithelial barrier dysfunction, inflammation, and coagulation predict higher mortality during treated HIV/AIDS.

[8] van Snick J (1990). Interleukin-6: an overview. *Annual Review of Immunology*.

[9] Marin B, Thiebaut R, Bucher HC (2009). Non-AIDS-defining deaths and immunodeficiency in the era of combination antiretroviral therapy. *AIDS*.

[10] Islam FM, Wu J, Jansson J, Wilson DP (2012). Relative risk of cardiovascular disease among people living with HIV: a systematic review and meta-analysis. *HIV Medicine*.

[11] Freiberg MS, Chang CC, Kuller LH (2013). HIV infection and the risk of acute myocardial infarction. *JAMA Internal Medicine*.

[12] Westhorpe CL, Maisa A, Spelman T (2014). Associations between surface markers on blood monocytes and carotid atherosclerosis in HIV-positive individuals. *Immunology and Cell Biology*.

[13] Davignon J, Ganz P (2004). Role of endothelial dysfunction in atherosclerosis. *Circulation*.

[14] Solages A, Vita JA, Thornton DJ (2006). Endothelial function in HIV-infected persons. *Clinical Infectious Diseases*.

[15] Tipping PG, Hancock WW (1993). Production of tumor necrosis factor and interleukin-1 by macrophages from human atheromatous plaques. *The American Journal of Pathology*.

[16] Rayment NB, Moss E, Faulkner L (1996). Synthesis of TNF*α* and TGF*β* mRNA in the different micro-environments within atheromatous plaques. *Cardiovascular Research*.

[17] Apostolopoulos J, Davenport P, Tipping PG (1996). Interleukin-8 production by macrophages from atheromatous plaques. *Arteriosclerosis, Thrombosis, and Vascular Biology*.

[18] Shah PK, Falk E, Badimon JJ (1995). Human monocyte-derived macrophages induce collagen breakdown in fibrous caps of atherosclerotic plaques: potential role of matrix-degrading metalloproteinases and implications for plaque rupture. *Circulation*.

[19] Rosenfeld ME, Yla-Herttuala S, Lipton BA, Ord VA, Witztum JL, Steinberg D (1992). Macrophage colony-stimulating factor mRNA and protein in atherosclerotic lesions of rabbits and humans. *The American Journal of Pathology*.

[20] Kruth HS (2001). Macrophage foam cells and atherosclerosis. *Frontiers in Bioscience*.

[21] Llodra J, Angeli V, Liu J, Trogan E, Fisher EA, Rendolph GJ (2004). Emigration of monocyte-derived cells from atherosclerotic lesions characterizes regressive, but not progressive, plaques. *Proceedings of the National Academy of Sciences of the United States of America*.

[22] Tabas I (2010). Macrophage death and defective inflammation resolution in atherosclerosis. *Nature Reviews Immunology*.

[23] Finn AV, Nakano M, Narula J, Kolodgie FD, Virmani R (2010). Concept of vulnerable/unstable plaque. *Arteriosclerosis, Thrombosis, and Vascular Biology*.

[24] Grundy SM, Cleeman JI, Merz CN (2004). Implications of recent clinical trials for the National Cholesterol Education Program Adult Treatment Panel III Guidelines. *Journal of the American College of Cardiology*.

[25] Riddler SA, Li X, Chu H (2007). Longitudinal changes in serum lipids among HIV-infected men on highly active antiretroviral therapy. *HIV Medicine*.

[26] Riddler SA, Smit E, Cole SR (2003). Impact of HIV infection and HAART on serum lipids in men. *The Journal of the American Medical Association*.

[27] Lake JE, Currier JS (2013). Metabolic disease in HIV infection. *The Lancet Infectious Diseases*.

[28] Fontas E, van Leth F, Sabin CA (2004). Lipid profiles in HIV-infected patients receiving combination antiretroviral therapy: are different antiretroviral drugs associated with different lipid profiles?. *The Journal of Infectious Diseases*.

[29] Friis-Moller N, Reiss P, Sabin CA (2007). Class of antiretroviral drugs and the risk of myocardial infarction. *The New England Journal of Medicine*.

[30] Mujawar Z, Rose H, Morrow MP (2006). Human immunodeficiency virus impairs reverse cholesterol transport from macrophages. *PLoS Biology*.

[31] Asztalos BF, Mujawar Z, Morrow MP (2010). Circulating nef induces dyslipidemia in simian immunodeficiency virus-infected macaques by suppressing cholesterol efflux. *The Journal of Infectious Diseases*.

[32] Aleman MM, Gardiner C, Harrison P, Wolberg AS (2011). Differential contributions of monocyte- and platelet-derived microparticles towards thrombin generation and fibrin formation and stability. *Journal of Thrombosis and Haemostasis*.

[33] Funderburg NT, Mayne E, Sieg SF (2010). Increased tissue factor expression on circulating monocytes in chronic HIV infection: relationship to in vivo coagulation and immune activation. *Blood*.

[34] Kuller LH, Tracy R, Belloso W (2008). Inflammatory and coagulation biomarkers and mortality in patients with HIV infection. *PLoS Medicine*.

[35] Funderburg NT, Zidar DA, Shive C (2012). Shared monocyte subset phenotypes in HIV-1 infection and in uninfected subjects with acute coronary syndrome. *Blood*.

[36] Tapp LD, Shantsila E, Wrigley BJ, Pamukcu B, Lip GYH (2012). The CD14++CD16+ monocyte subset and monocyte-platelet interactions in patients with ST-elevation myocardial infarction. *Journal of Thrombosis and Haemostasis*.

[37] Rudd JH, Warburton EA, Fryer TD (2002). Imaging atherosclerotic plaque inflammation with [^18^F]-fluorodeoxyglucose positron emission tomography. *Circulation*.

[38] Yarasheski KE, Laciny E, Overton ET (2012). ^18^FDG PET-CT imaging detects arterial inflammation and early atherosclerosis in HIV-infected adults with cardiovascular disease risk factors. *Journal of Inflammation*.

[39] Subramanian S, Tawakol A, Burdo TH (2012). Arterial inflammation in patients with HIV. *The Journal of the American Medical Association*.

[40] Anzinger JJ, Zhou J, Lam L Glut1 expression on intermediate monocytes is a potential marker of inflammation in HIV-positive subjects.

[41] Pedersen SF, Graebe M, Fisker Hag AM, Højgaard L, Sillesen H, Kjaer A (2010). Gene expression and 18FDG uptake in atherosclerotic carotid plaques. *Nuclear Medicine Communications*.

[42] Heaton RK, Clifford DB, Franklin DR (2010). HIV-associated neurocognitive disorders persist in the era of potent antiretroviral therapy: CHARTER Study. *Neurology*.

[43] Robertson KR, Smurzynski M, Parsons TD (2007). The prevalence and incidence of neurocognitive impairment in the HAART era. *AIDS*.

[44] Cysique LA, Brew BJ (2011). Prevalence of non-confounded HIV-associated neurocognitive impairment in the context of plasma HIV RNA suppression. *Journal of NeuroVirology*.

[45] Heaton RK, Franklin DR, Ellis RJ (2011). HIV-associated neurocognitive disorders before and during the era of combination antiretroviral therapy: differences in rates, nature, and predictors. *Journal of NeuroVirology*.

[46] Simioni S, Cavassini M, Annoni JM (2010). Cognitive dysfunction in HIV patients despite long-standing suppression of viremia. *AIDS*.

[47] Lucero C, Torres B, Leon A (2013). Rate and predictors of non-AIDS events in a cohort of HIV-infected patients with a CD4 T cell count above 500 cells/mm^3^. *AIDS Research and Human Retroviruses*.

[48] Wiley CA, Schrier RD, Nelson JA, Lampert PW, Oldstone MB (1986). Cellular localization of human immunodeficiency virus infection within the brains of acquired immune deficiency syndrome patients. *Proceedings of the National Academy of Sciences of the United States of America*.

[49] Williams KC, Corey S, Westmoreland SV (2001). Perivascular macrophages are the primary cell type productively infected by simian immunodeficiency virus in the brains of macaques: implications for the neuropathogenesis of AIDS. *The Journal of Experimental Medicine*.

[50] Bechmann I, Galea I, Perry VH (2007). What is the blood-brain barrier (not)?. *Trends in Immunology*.

[51] Reinhart TA, Rogan MJ, Huddleston D, Rausch DM, Eiden LE, Haase AT (1997). Simian immunodeficiency virus burden in tissues and cellular compartments during clinical latency and AIDS. *The Journal of Infectious Diseases*.

[52] Albright AV, Soldan SS, Gonzalez-Scarano F (2003). Pathogenesis of human immunodeficiency virus-induced neurological disease. *Journal of NeuroVirology*.

[53] Clay CC, Rodrigues DS, Ho YS (2007). Neuroinvasion of fluorescein-positive monocytes in acute simian immunodeficiency virus infection. *Journal of Virology*.

[54] Furtado MR, Callaway DS, Phair JP (1999). Persistence of HIV-1 transcription in peripheral-blood mononuclear cells in patients receiving potent antiretroviral therapy. *The New England Journal of Medicine*.

[55] Crowe SM, Sonza S (2000). HIV-1 can be recovered from a variety of cells including peripheral blood monocytes of patients receiving highly active antiretroviral therapy: a further obstacle to eradication. *Journal of Leukocyte Biology*.

[56] Sonza S, Mutimer HP, Oelrichs R (2001). Monocytes harbour replication-competent, non-latent HIV-1 in patients on highly active antiretroviral therapy. *AIDS*.

[57] Ginhoux F, Greter M, Leboeuf M (2010). Fate mapping analysis reveals that adult microglia derive from primitive macrophages. *Science*.

[58] Bechmann I, Priller J, Kovac A (2001). Immune surveillance of mouse brain perivascular spaces by blood-borne macrophages. *The European Journal of Neuroscience*.

[59] Kim WK, Alvarez X, Fisher J (2006). CD163 identifies perivascular macrophages in normal and viral encephalitic brains and potential precursors to perivascular macrophages in blood. *The American Journal of Pathology*.

[60] Valcour VG, Shiramizu BT, Sithinamsuwan P (2009). HIV DNA and cognition in a Thai longitudinal HAART initiation cohort: the SEARCH 001 Cohort study. *Neurology*.

[61] Shiramizu B, Ananworanich J, Chalermchai T (2012). Failure to clear intra-monocyte HIV infection linked to persistent neuropsychological testing impairment after first-line combined antiretroviral therapy. *Journal of NeuroVirology*.

[62] Ellery PJ, Tippett E, Chiu YL (2007). The CD16+ monocyte subset is more permissive to infection and preferentially harbors HIV-1 in vivo. *The Journal of Immunology*.

[63] Neuhaus J, Jacobs DR, Baker JV (2010). Markers of inflammation, coagulation, and renal function are elevated in adults with HIV infection. *The Journal of Infectious Diseases*.

[64] Murr C, Widner B, Wirleitner B, Fuchs D (2002). Neopterin as a marker for immune system activation. *Current Drug Metabolism*.

[65] Hoffmann G, Wirleitner B, Fuchs D (2003). Potential role of immune system activation-associated production of neopterin derivatives in humans. *Inflammation Research*.

[66] Edén A, Price RW, Spudich S, Fuchs D, Hagberg L, Gisslén M (2007). Immune activation of the central nervous system is still present after >4 years of effective highly active antiretroviral therapy. *The Journal of Infectious Diseases*.

[67] Giulian D, Vaca K, Noonan CA (1990). Secretion of neurotoxins by mononuclear phagocytes infected with HIV-1. *Science*.

[68] Giulian D, Wendt E, Vaca K, Noonan CA (1993). The envelope glycoprotein of human immunodeficiency virus type 1 stimulates release of neurotoxins from monocytes. *Proceedings of the National Academy of Sciences of the United States of America*.

[69] Bonfoco E, Krainc D, Ankarcrona M, Nicotera P, Lipton SA (1995). Apoptosis and necrosis: two distinct events induced, respectively, by mild and intense insults with N-methyl-D-aspartate or nitric oxide/superoxide in cortical cell cultures. *Proceedings of the National Academy of Sciences of the United States of America*.

[70] Meucci O, Fatatis A, Simen AA, Bushell TJ, Gray PW, Miller RJ (1998). Chemokines regulate hippocampal neuronal signaling and gp120 neurotoxicity. *Proceedings of the National Academy of Sciences of the United States of America*.

[71] Piller SC, Jans P, Gage PW, Jans DA (1998). Extracellular HIV-1 virus protein R causes a large inward current and cell death in cultured hippocampal neurons: implications for AIDS pathology. *Proceedings of the National Academy of Sciences of the United States of America*.

[72] Liu Y, Jones M, Hingtgen CM (2000). Uptake of HIV-1 tat protein mediated by low-density lipoprotein receptor-related protein disrupts the neuronal metabolic balance of the receptor ligands. *Nature Medicine*.

[73] Martin GE, Gouillou M, Hearps AC (2013). Age-associated changes in monocyte and innate immune activation markers occur more rapidly in HIV infected women. *PLoS ONE*.

[74] Hearps AC, Maisa A, Cheng WJ (2012). HIV infection induces age-related changes to monocytes and innate immune activation in young men that persist despite combination antiretroviral therapy. *AIDS*.

[75] Kamat A, Lyons JL, Misra V (2012). Monocyte activation markers in cerebrospinal fluid associated with impaired neurocognitive testing in advanced HIV infection. *Journal of Acquired Immune Deficiency Syndromes*.

[76] Jalbert E, Crawford TQ, D’Antoni ML (2013). IL-1Beta enriched monocytes mount massive IL-6 responses to common inflammatory triggers among chronically HIV-1 infected adults on stable anti-retroviral therapy at risk for cardiovascular disease. *PLoS ONE*.

[77] Duprez DA, Neuhaus J, Kuller LH (2012). Inflammation, coagulation and cardiovascular disease in HIV-infected individuals. *PLoS ONE*.

[78] Airoldi M, Bandera A, Trabattoni D (2012). Neurocognitive impairment in HIV-infected naïve patients with advanced disease: the role of virus and intrathecal immune activation. *Clinical and Developmental Immunology*.

[79] Ershler WB, Sun WH, Binkley N (1993). Interleukin-6 and aging: blood levels and mononuclear cell production increase with advancing age and in vitro production is modifiable by dietary restriction. *Lymphokine and Cytokine Research*.

[80] Heinrich PC, Castell JV, Andus T (1990). Interleukin-6 and the acute phase response. *The Biochemical Journal*.

[81] Trzonkowski P, Myśliwska J, Pawelec G, Myśliwski A (2009). From bench to bedside and back: the SENIEUR Protocol and the efficacy of influenza vaccination in the elderly. *Biogerontology*.

[82] Bazil V, Strominger JL (1991). Shedding as a mechanism of down-modulation of CD14 on stimulated human monocytes. *The Journal of Immunology*.

[83] Lien E, Aukrust P, Sundan A, Müller F, Frøland SS, Espevik T (1998). Elevated levels of serum-soluble CD14 in human immunodeficiency virus type 1 (HIV-1) infection: correlation to disease progression and clinical events. *Blood*.

[84] Méndez-Lagares G, Romero-Sánchez MC, Ruiz-Mateos E (2013). Long-term suppressive combined antiretroviral treatment does not normalize the serum level of soluble CD14. *The Journal of Infectious Diseases*.

[85] Sandler NG, Wand H, Roque A (2011). Plasma levels of soluble CD14 independently predict mortality in HIV infection. *The Journal of Infectious Diseases*.

[86] Kelesidis T, Kendall MA, Yang OO, Hodis HN, Currier JS (2012). Biomarkers of microbial translocation and macrophage activation: association with progression of subclinical atherosclerosis in HIV-1 infection. *The Journal of Infectious Diseases*.

[87] Bas S, Gauthier BR, Spenato U, Stingelin S, Gabay C (2004). CD14 is an acute-phase protein. *The Journal of Immunology*.

[88] Moller HJ (2012). Soluble CD163. *Scandinavian Journal of Clinical and Laboratory Investigation*.

[89] Burdo TH, Lentz MR, Autissier P (2011). Soluble CD163 made by monocyte/macrophages is a novel marker of HIV activity in early and chronic infection prior to and after antiretroviral therapy. *The Journal of Infectious Diseases*.

[90] Burdo TH, Lo J, Abbara S (2011). Soluble CD163, a novel marker of activated macrophages, is elevated and associated with noncalcified coronary plaque in HIV-infected patients. *The Journal of Infectious Diseases*.

[91] Tabib A, Leroux C, Mornex JF, Loire R (2000). Accelerated coronary atherosclerosis and arteriosclerosis in young human-immunodeficiency-virus-positive patients. *Coronary Artery Disease*.

[92] Burdo TH, Weiffenbach A, Woods SP, Letendre S, Ellis RJ, Williams KC (2013). Elevated sCD163 in plasma but not cerebrospinal fluid is a marker of neurocognitive impairment in HIV infection. *AIDS*.

[93] Brenchley JM, Price DA, Schacker TW (2006). Microbial translocation is a cause of systemic immune activation in chronic HIV infection. *Nature Medicine*.

[94] Brenchley JM, Schacker TW, Ruff LE (2004). CD4+ T cell depletion during all stages of HIV disease occurs predominantly in the gastrointestinal tract. *The Journal of Experimental Medicine*.

[95] Levels JH, Abraham PR, van den Ende A, van Deventer SJH (2001). Distribution and kinetics of lipoprotein-bound endotoxin. *Infection and Immunity*.

[96] Flegel WA, Wolpl A, Mannel DN, Northoff H (1989). Inhibition of endotoxin-induced activation of human monocytes by human lipoproteins. *Infection and Immunity*.

[97] Birjmohun RS, van Leuven SI, Levels JH (2007). High-density lipoprotein attenuates inflammation and coagulation response on endotoxin challenge in humans. *Arteriosclerosis, Thrombosis, and Vascular Biology*.

[98] Anastos K, Lu D, Shi Q (2007). Association of serum lipid levels with HIV serostatus, specific antiretroviral agents, and treatment regimens. *Journal of Acquired Immune Deficiency Syndromes*.

[99] Allers K, Fehr M, Conrad K (2013). Macrophages accumulate in the gut mucosa of untreated HIV-infected patients. *The Journal of Infectious Diseases*.

[100] El-Sadr WM, Lundgren J, Neaton JD (2006). CD4+ count-guided interruption of antiretroviral treatment. *The New England Journal of Medicine*.

[101] Palmer S, Wiegand AP, Maldarelli F (2003). New real-time reverse transcriptase-initiated PCR assay with single-copy sensitivity for human immunodeficiency virus type 1 RNA in plasma. *Journal of Clinical Microbiology*.

[102] Palmer S, Maldarelli F, Wiegand A (2008). Low-level viremia persists for at least 7 years in patients on suppressive antiretroviral therapy. *Proceedings of the National Academy of Sciences of the United States of America*.

[103] Maldarelli F, Palmer S, King MS (2007). ART suppresses plasma HIV-1 RNA to a stable set point predicted by pretherapy viremia. *PLoS Pathogens*.

[104] Hatano H, Strain MC, Scherzer R (2013). Increase in 2-long terminal repeat circles and decrease in D-dimer after raltegravir intensification in patients with treated HIV infection: a randomized, placebo-controlled trial. *The Journal of Infectious Diseases*.

[105] Hatano H, Hayes TL, Dahl V (2011). A randomized, controlled trial of raltegravir intensification in antiretroviral-treated, HIV-infected patients with a suboptimal CD4+ T cell response. *The Journal of Infectious Diseases*.

[106] Palmer S (2013). Advances in detection and monitoring of plasma viremia in HIV-infected individuals receiving antiretroviral therapy. *Current Opinion in HIV and AIDS*.

[107] Marini E, Tiberio L, Caracciolo S (2008). HIV-1 matrix protein p17 binds to monocytes and selectively stimulates MCP-1 secretion: role of transcriptional factor AP-1. *Cellular Microbiology*.

[108] Wahl LM, Corcoran ML, Pyle SW, Arthur LO, Harel-Bellan A, Farrar WL (1989). Human immunodeficiency virus glycoprotein (gp120) induction of monocyte arachidonic acid metabolites and interleukin 1. *Proceedings of the National Academy of Sciences of the United States of America*.

[109] Clouse KA, Cosentino LM, Weih KA (1991). The HIV-1 gp120 envelope protein has the intrinsic capacity to stimulate monokine secretion. *The Journal of Immunology*.

[110] Jackson JB, Erice A, Englund JA, Edson JR, Balfour HH (1988). Prevalence of cytomegalovirus antibody in hemophiliacs and homosexuals infected with human immunodeficiency virus type 1. *Transfusion*.

[111] Verbraak FD, Boom R, Wertheim-van Dillen PM, Van den Horn GJ, Kijlstra A, de Smet MD (1999). Influence of highly active antiretroviral therapy on the development of CMV disease in HIV positive patients at high risk for CMV disease. *The British Journal of Ophthalmology*.

[112] Naeger DM, Martin JN, Sinclair E (2010). Cytomegalovirus-specific T cells persist at very high levels during long-term antiretroviral treatment of HIV disease. *PLoS ONE*.

[113] Rice GP, Schrier RD, Oldstone MB (1984). Cytomegalovirus infects human lymphocytes and monocytes: virus expression is restricted to immediate-early gene products. *Proceedings of the National Academy of Sciences of the United States of America*.

[114] Soderberg-Naucler C, Streblow DN, Fish KN, Allan-Yorke J, Smith PP, Nelson JA (2001). Reactivation of latent human cytomegalovirus in CD14+ monocytes is differentiation dependent. *Journal of Virology*.

[115] Yurochko AD, Huang ES (1999). Human cytomegalovirus binding to human monocytes induces immunoregulatory gene expression. *The Journal of Immunology*.

[116] Chan G, Bivins-Smith ER, Smith MS, Yurochko AD (2008). Transcriptome analysis of NF-*κ*B- and phosphatidylinositol 3-kinase-regulated genes in human cytomegalovirus-infected monocytes. *Journal of Virology*.

[117] Miller CS, Berger JR, Mootoor Y, Avdiushko SA, Zhu H, Kryscio RJ (2006). High prevalence of multiple human herpesviruses in saliva from human immunodeficiency virus-infected persons in the era of highly active antiretroviral therapy. *Journal of Clinical Microbiology*.

[118] Savard M, Belanger C, Tardif M, Gourde P, Flamand L, Gosselin J (2000). Infection of primary human monocytes by Epstein-Barr virus. *Journal of Virology*.

[119] Gaudreault E, Fiola S, Olivier M, Gosselin J (2007). Epstein-Barr virus induces MCP-1 secretion by human monocytes via TLR2. *Journal of Virology*.

[120] Rappocciolo G, Jenkins FJ, Hensler HR (2006). DC-SIGN is a receptor for human herpesvirus 8 on dendritic cells and macrophages. *The Journal of Immunology*.

[121] Blasig C, Zietz C, Haar B (1997). Monocytes in Kaposi’s sarcoma lesions are productively infected by human herpesvirus 8. *Journal of Virology*.

[122] Monini P, Colombini S, Sturzl M (1999). Reactivation and persistence of human herpesvirus-8 infection in B cells and monocytes by Th-1 cytokines increased in Kaposi’s sarcoma. *Blood*.

[123] West J, Damania B (2008). Upregulation of the TLR3 pathway by Kaposi’s sarcoma-associated herpesvirus during primary infection. *Journal of Virology*.

[124] Ahmad R, El Bassam S, Cordeiro P, Menezes J (2008). Requirement of TLR2-mediated signaling for the induction of IL-15 gene expression in human monocytic cells by HSV-1. *Blood*.

[125] Melchjorsen J, Siren J, Julkunen I, Paludan SR, Matikainen S (2006). Induction of cytokine expression by herpes simplex virus in human monocyte-derived macrophages and dendritic cells is dependent on virus replication and is counteracted by ICP27 targeting NF-*κ*B and IRF-3. *Journal of General Virology*.

